# Convolutional neural networks can accurately distinguish four histologic growth patterns of lung adenocarcinoma in digital slides

**DOI:** 10.1038/s41598-018-37638-9

**Published:** 2019-02-06

**Authors:** Arkadiusz Gertych, Zaneta Swiderska-Chadaj, Zhaoxuan Ma, Nathan Ing, Tomasz Markiewicz, Szczepan Cierniak, Hootan Salemi, Samuel Guzman, Ann E. Walts, Beatrice S. Knudsen

**Affiliations:** 1 Department of Surgery, Cedars-Sinai Medical Center, Los Angeles, California USA; 2 Department of Pathology and Laboratory Medicine, Cedars-Sinai Medical Center, Los Angeles, California USA; 3 Faculty of Electrical Engineering, Warsaw University of Technology, Warsaw, Poland; 4 Department of Biomedical Sciences, Cedars-Sinai Medical Center, Los Angeles, California USA; 5 Department of Pathology, Military Institute of Medicine, Warsaw, Poland

## Abstract

During the diagnostic workup of lung adenocarcinomas (LAC), pathologists evaluate distinct histological tumor growth patterns. The percentage of each pattern on multiple slides bears prognostic significance. To assist with the quantification of growth patterns, we constructed a pipeline equipped with a convolutional neural network (CNN) and soft-voting as the decision function to recognize solid, micropapillary, acinar, and cribriform growth patterns, and non-tumor areas. Slides of primary LAC were obtained from Cedars-Sinai Medical Center (CSMC), the Military Institute of Medicine in Warsaw and the TCGA portal. Several CNN models trained with 19,924 image tiles extracted from 78 slides (MIMW and CSMC) were evaluated on 128 test slides from the three sites by F1-score and accuracy using manual tumor annotations by pathologist. The best CNN yielded F1-scores of 0.91 (solid), 0.76 (micropapillary), 0.74 (acinar), 0.6 (cribriform), and 0.96 (non-tumor) respectively. The overall accuracy of distinguishing the five tissue classes was 89.24%. Slide-based accuracy in the CSMC set (88.5%) was significantly better (*p* < 2.3E-4) than the accuracy in the MIMW (84.2%) and TCGA (84%) sets due to superior slide quality. Our model can work side-by-side with a pathologist to accurately quantify the percentages of growth patterns in tumors with mixed LAC patterns.

## Introduction

Lung cancer is currently the second most common cancer in men and women and the leading cause of cancer-related deaths worldwide. Within invasive lung adenocarcinomas (LAC), the new WHO classification separates six histological patterns: lepidic, papillary, micropapillary, acinar, cribriform and solid and recommends that surgically excised tumors be subclassified based on the predominant growth pattern^1^. Based on this recommendation, the histologic patterns observed in the tumor are quantified in 5% increments and reported.

In addition to the tumor stage at diagnosis, the predominant tumor growth pattern impacts prognosis^2–4^. While tumors with mostly lepidic and acinar histology tend to be less aggressive^5,6^, tumors with predominantly micropapillary and solid patterns have been consistently associated with poorer prognosis^7–9^. Recently, the percentage of cribriform pattern has also been identified as a marker of unfavorable prognosis^10,11^. Over 80% of LACs demonstrate a mixture of two or more histologic growth patterns, and the evaluation of tumor histology requires a composite manual estimation of the percentage of each pattern in each of several slides prepared from the tumor. The subjectivity inherent in such estimations contributes to only modest agreement between pathologists in assessing growth patterns of LAC^12,13^.

Machine learning approaches have been shown to improve the accuracy and automation of histopathologic slide analysis^14^. Convolutional neural networks (CNNs) are currently the state-of-the-art generation of tools to build decision-making workflows in digital pathology. When presented with sufficient annotated training image data, CNNs can learn complex histological patterns from images through a deconvolution of the image content into thousands of salient features followed by selection and aggregation of the most meaningful features and then recognize these patterns in as yet unseen images. Applications involving CNNs in digital pathology are numerous and range from the recognition of tumor regions to the extraction of “hidden” tumor characteristics for biomarker development^15,16^.

CNNs can also be instrumental to systematically analyze lung tumors whose histomorphologic heterogeneity poses a challenge to direct visual microscopic quantification of growth patterns by pathologists.

Currently available computer-assisted methods for the analysis of slides with lung tumors focus on the classification of one or two types of lung cancer and separation of tumor from non-tumor. In the study by Luo *et al*., hand-crafted image features extracted from squared image tiles were used to distinguish areas of adenocarcinoma from squamous carcinoma of the lung^17^. These features were used in statistical models to predict survival in cases from The Cancer Genome Atlas (TCGA). Using digital histology slides from the National Lung Screening Trial repository^18^, Wang *et al*. trained a CNN to delineate tumor, and prognosticated patient survival outcome from tumor shape^19^. Their tool assigned consecutive square image tiles to tumor, background or non-tumor categories. Other CNN models were trained to distinguish adenocarcinoma from small cell carcinoma^20,21^, or adenocarcinoma from squamous carcinoma and  from non-tumor tissue^22^. Furthermore, using whole slides of LAC, a CNN was trained to predict the presence of six gene mutations based on associated morphologic tumor features^22^. These and other studies demonstrate the feasibility of using CNNs for histologic analysis of lung cancer.

Primary lung adenocarcinomas are heterogeneous tumors which commonly exhibit a mixture of different histologic growth patterns and molecular profiles that are distinct from those of squamous cell carcinoma^23^. Besides a proof-of-concept study by our group demonstrating the ability of a machine learning approach to distinguish solid and micropapillary growth patterns in digital images of LAC^24^, there is a lack of computer-assisted methods to aid pathologists in the comprehensive quantification of growth patterns of LAC. In this study, we developed a pipeline to distinguish four growth patterns of pulmonary adenocarcinoma (acinar, micropapillary, solid, and cribriform) and separate tumor regions from non-tumor. We focused on light-weight CNN architectures and strategies that have low hardware requirements and compared two CNN architectures (pre-trained and de-novo trained) to assess their performances in classification of these five tissue classes. Our models were validated using digital slides from 3 independent cohorts.

## Materials

### Ethics Statement

Data collection and analysis for this research project was approved by the Office of Research Compliance at the Cedars-Sinai Medical Center (approval # Pro00051794) and the Research Ethics Board of the Military Institute of Medicine in Poland (Number: 30/WIM/2016). Prior to obtaining digital slides for analysis, all glass slides were de-identified to comply with HIPAA regulations, and the analysis of digital slides was conducted in accordance with approved guidelines at both intuitions.

### Cohorts and data collection

**Figure 1:**
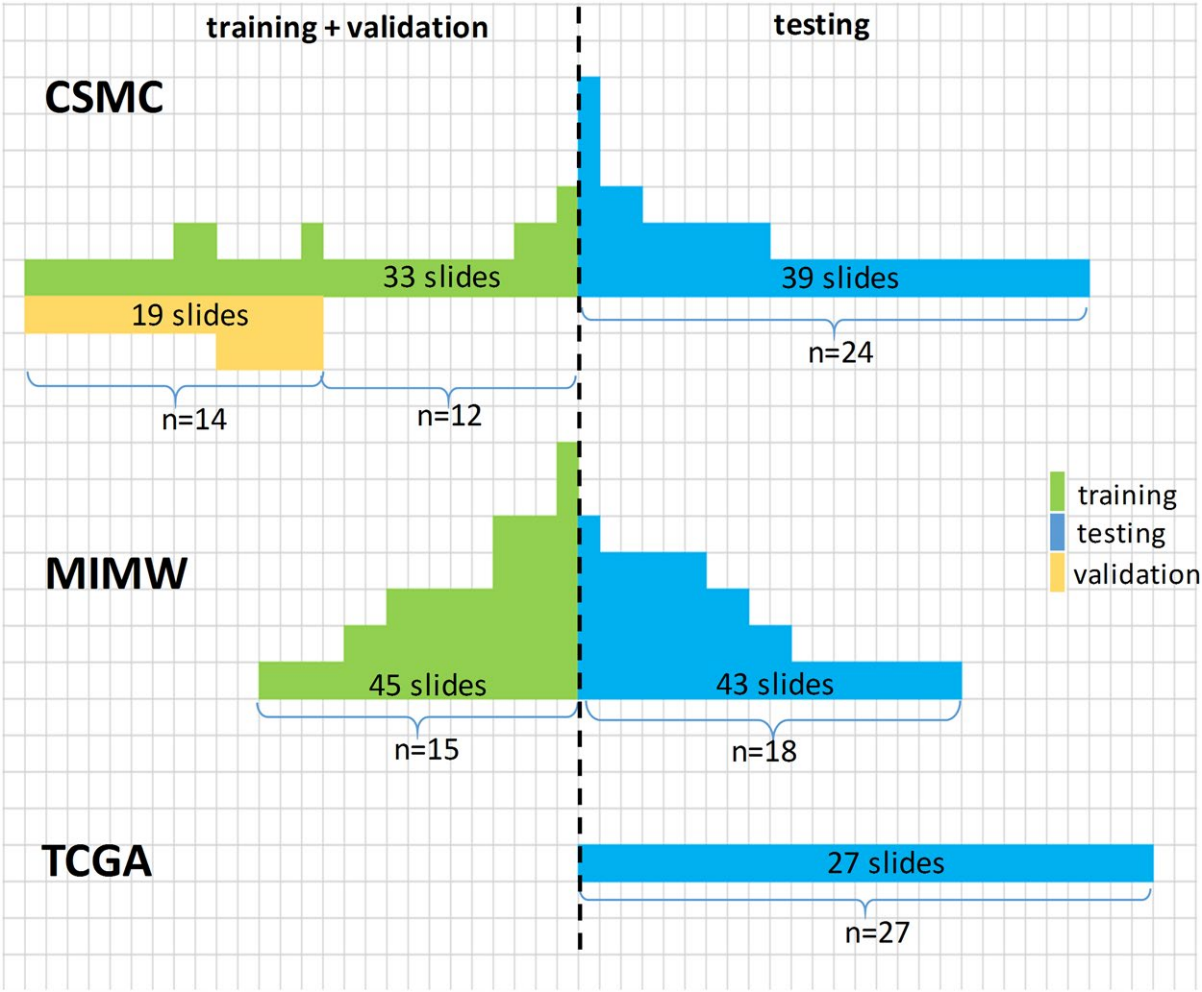
A breakdown of CSMC, MIMW and TCGA cases into training, validation and testing cohorts. The number of slides per case varies from 1 to 7. Each slide was obtained from a different paraffin block.

This study involved digital slides from three cohorts. Hematoxylin and eosin (H&E) stained slides from cases previously diagnosed as primary lung adenocarcinoma were retrieved from pathology department archives at Cedars-Sinai Medical Center in Los Angeles (CSMC, 50 cases) and the Military Institute of Medicine in Warsaw, Poland (MIMW, 33 cases). The third cohort consists of 27 digital slides (one slide per case) identified using “primary tumor” and “lung adenocarcinoma” as key search terms and downloaded from the TCGA portal. Prior to inclusion in the study, slides in the CSMC and MIMW cohorts underwent manual review (AW, SG at CSMC; SC at MIMW) for tissue quality and to confirm the presence of at least one of the following tumor growth patterns: acinar, micropapillary, solid or cribriform in each slide. Slides with other growth patterns were excluded. After downloading, digital slides from the TCGA portal were reviewed (AW) in the same manner. No preference other than tumor growth pattern was given to cases during the selection. For most of the cases only one slide was available. Other cases had multiple slides: up to 7 in the MIMW cohort, and up to 6 in the CSMC cohort, with each slide prepared from a different formalin fixed paraffin embedded section of the LAC. Cases from the CSMC and the MIMW cohorts were randomly partitioned into training and validation sets. We randomly picked a subset of 19 slides from the CSMC training cases, to validate our CNN models. A detailed breakdown of the datasets constructed from the CSMC, MIMW and TCGA cohorts is shown in Fig. 1.

### Slide digitization

CSMC slides were digitized using Aperio AT Turbo (Leica Biosystems, Vista, CA), whereas slides from MIMW were digitized with Pannoramic 250 Flash II (3DHISTECH, Budapest, Hungary) whole slide scanner. Digital slides deposited in TCGA had been obtained through scanning with Aperio AT Turbo at either x20 or x40 magnification. Each digital slide was encoded as a set of multiresolution 24 bit RGB matrices and saved in SVS (slides from CSMC and TCGA) or MRXS (slides from MIMW) image format. Due to differences in hardware configurations between the Aperio and Pannoramic scanners and scanning modes set at the time of slide scanning, the pixel size and magnification for SVS and MRXS formats varied (Supplementary Table 1). Digital slides from MIMW and CSMC were manually checked for blur artifacts, and affected slides were rescanned prior to downstream processing.

### Ground truth annotations

**Figure 2:**
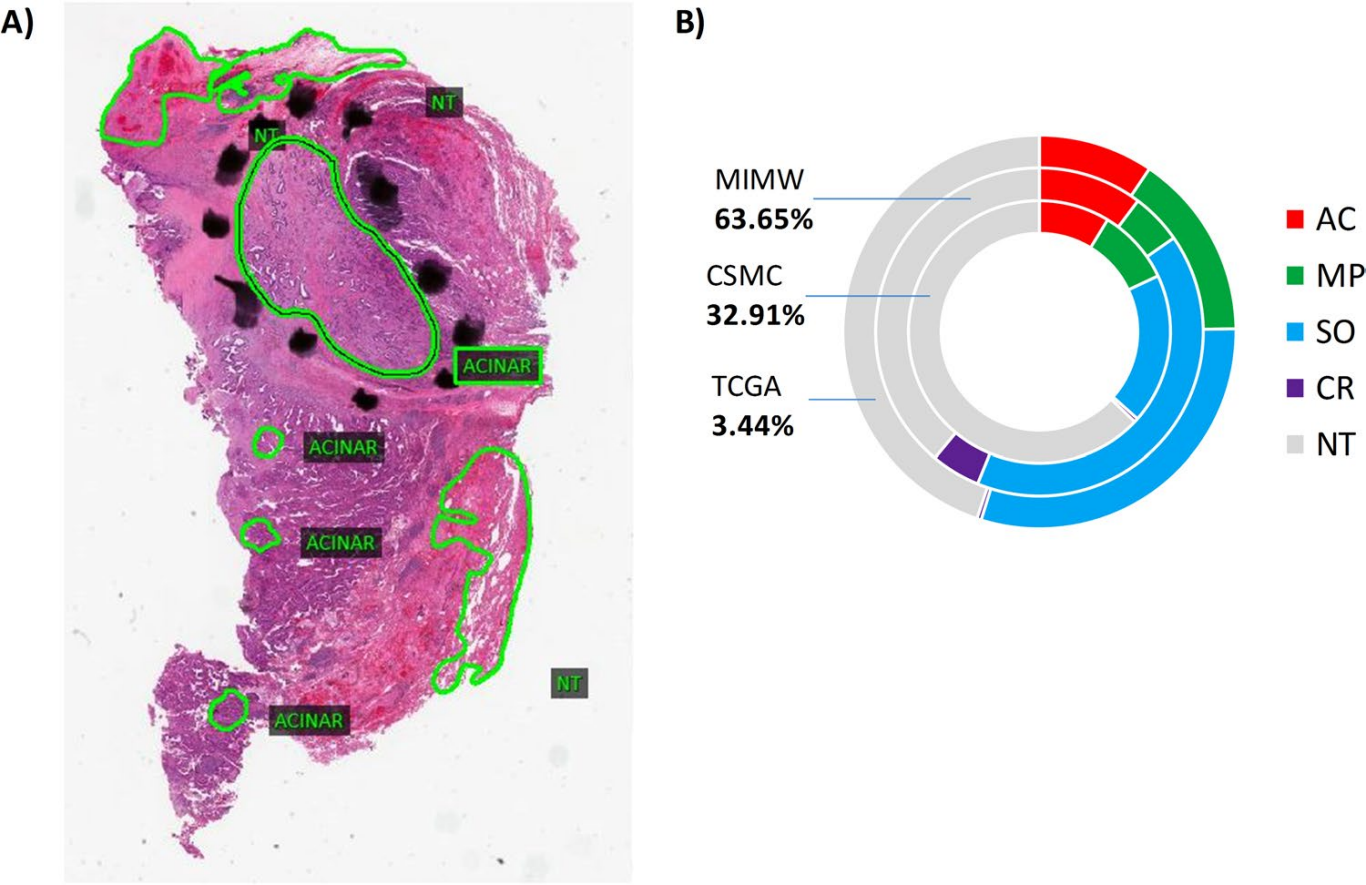
Ground truth annotations in validation and test (n = 128) slides: (**A**) example of manual delineations (green line) and labeling performed by a pathologist using Aperio digital slide viewer. Pixels under each annotation were sorted into five tissue classes and then counted to reflect proportions shown in (**B**). The total number of annotated pixels originating from tumors is closely matched by the number of pixels from non-tumor areas (53% to 47%). Solid growth patterns comprised 62% of total tumor pixels. 18.3% of the tumor pixels were of acinar, 12.8% of microcapillary and 6.1% of cribriform growth. Most of the annotations come from the MIMW (43 slides) while the CSMC and TCGA contributed 58 and 27 slides, respectively.

Pathologists from CSMC provided ground truth annotations of the four patterns of LAC and non-tumor areas directly on digital slides using Aperio ImageScope viewer (ver. 12.3, Leica Biosystems, Vista, CA). The pathologists first inspected the slides at 10x or higher magnification to identify areas of tumor, then reduced the magnification in order to circle and label areas of tumor as acinar, solid, micropapillary or cribriform growth pattern. Regions composed entirely of non-tumorous components including alveoli, stroma, clusters of immune cells, bronchial cartilage and epithelium, blood vessels, or their admixtures were collectively labeled as non-tumor. On each digital slide, a pathologist traced 1–10 tumor and up to 3 non-tumor areas. Since accurate tracing of tumor borders was very time consuming, the areas for annotation were arbitrarily selected without preference to the specific tumor growth patterns, or the size, shape or location of the annotated areas. Prior to annotation, the cases were divided into training and test sets as described above. In total, outlines from 206 digital slides (110 cases) (Fig. 1, Supplementary Table 1) were exported through the viewer and served as ground truth tissue masks to train or test classification models. Annotations in test and validation slides are detailed in Fig. 2 and Supplementary Table 2.

### Image tiles to train CNN models

**Figure 3:**
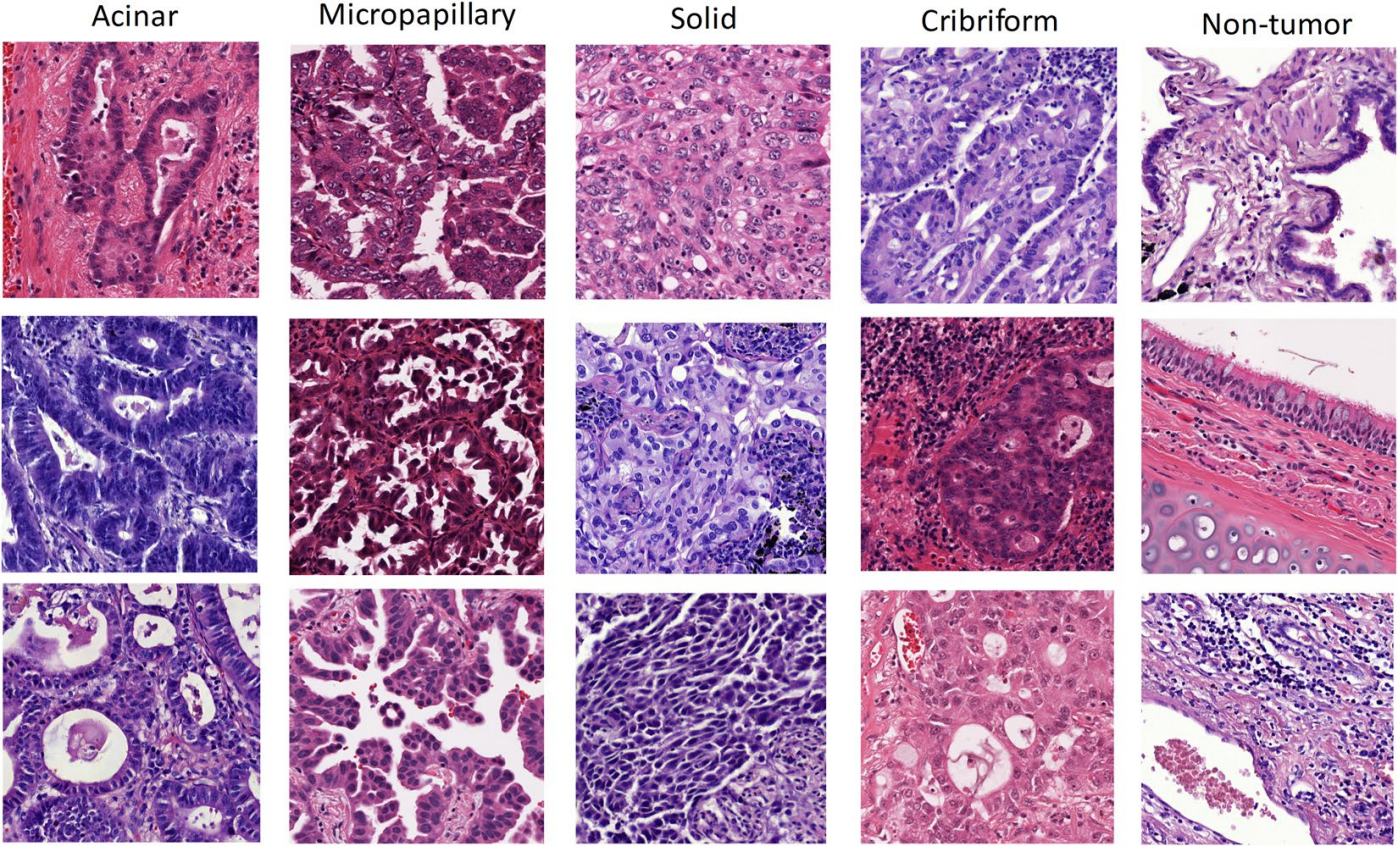
Example image tiles of LAC tumor growth patterns used for CNN training extracted from MIMW and CSMC training slides. Tiles with dark purple/blue straining (one each of solid, acinar and cribriform) are from MIMW training slides. Other tiles are from CSMC slides.

Areas on digital slides underneath the ground truth masks were randomly sampled to extract adjacent and non-overlapping square image tiles. Pre-screened tiles that contained clearly readable and high-quality tissue areas were reviewed by the pathologist (AW). A tile with tumor was considered suitable for CNN training if the pathologist could assess the growth pattern based on the tumor architecture in the tile. Tumor tiles were labeled as AC (acinar), MP (micropapillary), SO (solid), or CR (cribriform) (Fig. 3). Tiles in which the pathologist could not definitively assign a tumor growth pattern were excluded. Tiles without cancer cells were labeled as non-tumor (NT). All tiles were extracted from full resolution digital slides. Prior to extracting tiles, our team determined the minimal tile size sufficient for the pathologist to assess tumor growth patterns. The tile size was chosen to ensure resolution of nuclear features and include cell organization and larger structures that comprised the tumor growth pattern. A tile size of 600 × 600 pixels (CSMC cases were scanned at 20x) – an equivalent of 9 × 10^−3^mm^2^ area was found optimal. Depending on scanning magnification, the size of tiles extracted from MIMW and TCGA slides (Supplementary Table 1) was adjusted to match this area, yielding 19,942 image tiles for CNN training (Table 1).

**Table 1:** Summary of image tiles from CSMC and MIMW training cohorts.

Image tiles	CSMC	MIMW	Total
ACINAR	1,533	2,670	4,203
MICROPAPILLARY	2,071	1,165	3,236
SOLID	2,357	1,205	3,562
CRIBIFORM	863	2,375	3,238
NON-TUMOR	3,480	2,223	5,703
Total	10,304	9,638	**19**,**942**

## Methods

### Image tiles augmentation

Training of a CNN for a multiclass classification task requires thousands of training images^25^. However, collecting image data originating from manual input is costly and time consuming. Image augmentation reduces the effort needed to acquire additional training data, improves the robustness and ability of CNN to generalize, and decreases the risk of overfitting^26^. It relies on existing images whose content is manipulated to create multiple altered copies of a single image within parameters determined by the desired task. In our approach, augmentation is a product of color and image orientation alteration. Color augmentation is justified to sample from the range of hues (Fig. 3), that result from inter- and intra-laboratory variance in H&E staining. Orientation augmentation is justified since we aim to identify histologic growth patterns with no inherent orientation with respect to imaging, and which may naturally occur in any orientation. The color alteration is accomplished by modifying the original image coloration according to four new color patterns with the goal to simulate variations in H&E staining. We began by extracting *Lab* color components from the training tiles that we then grouped into four clusters using k-means clustering algorithm. Next, we calculated the mean and standard deviation of *L*, *a* and *b* color components in a cluster to define one new target *Lab* color pattern. In total, four color patterns were obtained by separately clustering *Lab* color components from CSMC and MIMW training tiles. These four color patterns were subsequently used in the Reinhard’s color transfer technique^27^ to give an original image tile four new appearances. The original image and each of the four color transformed image tiles were then rotated by 0, 90, 180 and 270 degrees, then diagonally flipped, rotated again, and saved after each rotation or flipping (Supplementary Fig. 1). By implementing the color and orientation augmentation, the original set of tiles was expanded to 797,680 image tiles for training.

### CNN training

The progress of deep learning technologies has led to the development of numerous CNN architectures. For our project, we trained and tested publicly available GoogLeNet^28^, ResNet-50^29^ and AlexNet^30^ CNNs that have been shown useful in pattern recognition tasks pertaining to digital pathology. Although AlexNet has competitors such as GoogLeNet, Inception-v3, or ResNet-50 that seem to outperform it in selected applications, we followed previously published works^31–34^ and incorporated this model into our study for its high generalization capability and low memory footprint during inference.

As AlexNet has not been used previously to classify growth patterns in heterogenous tumors, we elected to test a model that was pretrained on LSVRC-2010 ImageNet database with 1000 different classes of natural objects^30^ and was fine-tuned (FT) using our data (FT-AlexNet). Our training data were also used to train AlexNet with de-novo initialized weights (DN-AlexNet). The augmented tiles were downsized to 256 × 256 pixels for CNN training. The FT was performed in Caffe environment^35^ by training for 205,000 iterations using 80% of image tiles randomly picked from the augmented set and tested while being trained on the remining 20% of tiles. The learning rate, gamma, and momentum for stochastic gradient descent (SGD) optimizer were set to 0.01, 0.9 and 0.1 respectively. The DN-AlexNet was trained using MatConvNet plugin for Matlab (ver. 2017a, Mathworks Natic, MA) with learning rate logarithmically decreasing from 0.1 to 0.001 over 20 epochs. GoogLeNet was also trained in Caffe for 205,000 iterations with 90%/10% ratio of image tiles randomly picked for training/testing. The optimization was performed using the SGD optimizer with momentum set to 0.9, learning rate 0.001, and gamma 0.95. ResNet-50 was trained on the MatConvNet^36^ platform in Matlab for 90 epochs with a batch size of 256 images. The learning rate for SGD was initially set to 0.1 and then multiplied by 1/10 every 30 epochs, and momentum was 0.9. ResNet-50 and DN-AlexNet were trained using all available training data. Dropout^37^, at a 0.5 rate was applied to hidden layers during training. All CNNs were trained with batch-normalized images. After random weights initialization, DN-AlexNet was trained de novo four times, Resnet-50 and GoogLeNet were each trained three times, and FT-AlexNet was trained once. All models were tested to determine their fitness for identifying growth patterns of LAC and to identify the best performing model.

**Figure 4:**
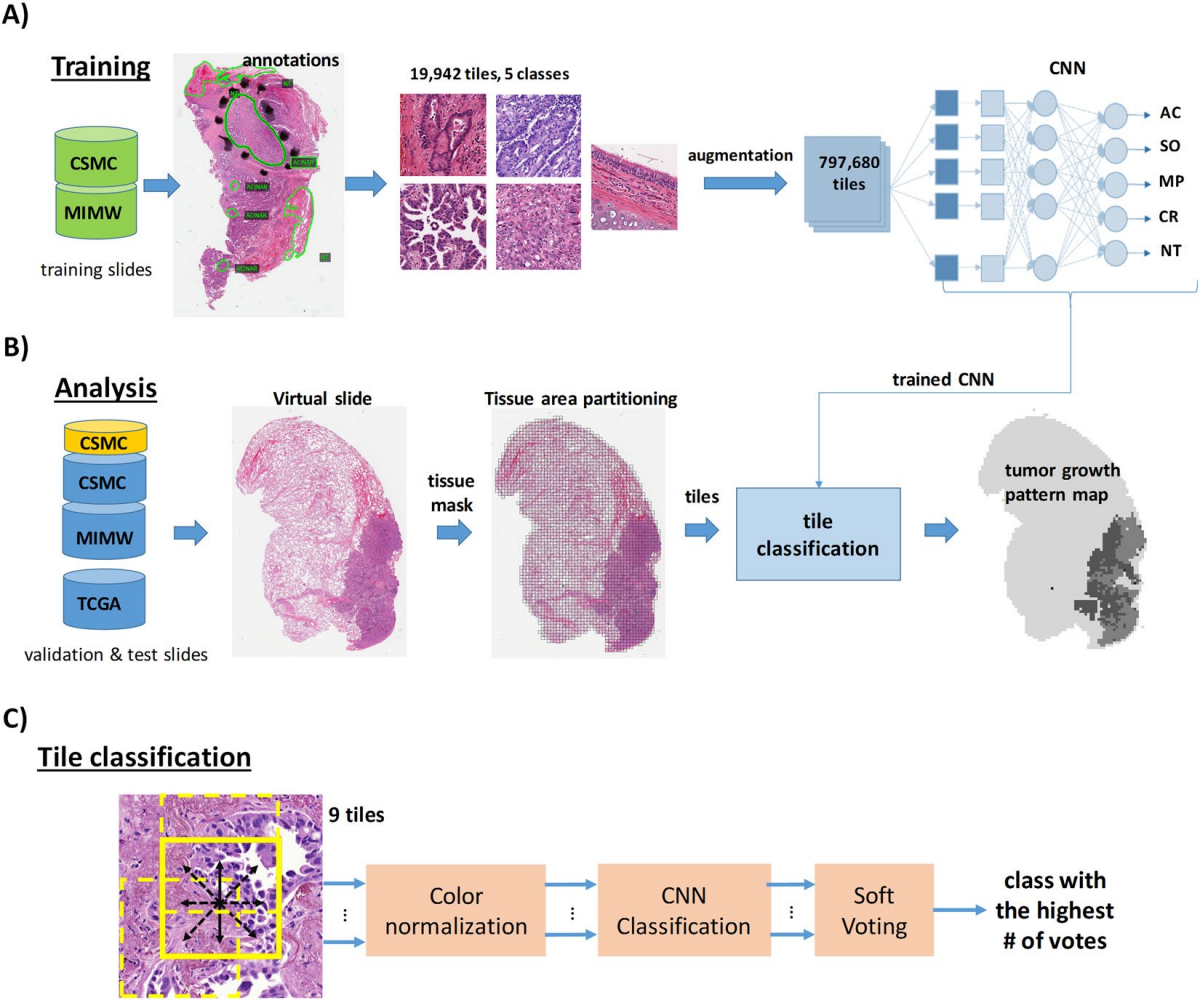
Overview of the WSI analysis pipeline: CNN trained with an augmented set of images from the training slides (**A**), is applied to classify tiles from test and validation slides (**B**) in a contextual (soft voting) manner (**C**). Eight overlapping tiles generated by respectively shifting the central tile by 1/3 of its length horizontally, vertically or diagonally to the central tile, and the central tile are independently classified by the CNN. The final classification result, which is the most frequently detected class (either AC, SO, MP, CR, or NT) amongst these nine tiles is then assigned to the central tile.

The FT-AlexNet was considered a baseline for the de-novo trained models. All models were trained on Nvidia GPUs. After training, the models were plugged into our WSI processing pipeline to evaluate performance (Fig. 4A).

### Digital slide processing pipeline

Our processing pipeline consists of three parts: 1) foreground tissue localization and partitioning into tiles, 2) individual tile classification by CNN, and 3) a module outputting tumor maps from classified WSIs (Fig. 4). To reduce the overall WSI analysis time, a tissue masking algorithm first located tissue area as foreground against white optical background by applying an intensity threshold (t = 230) to a gray level WSI image at low magnification (5x). Subsequently, the tissue mask was refined by hole filling and morphological closing. A grid of square tiles was then overlaid onto the mask. Tiles with insufficient tissue pixels (<20% of white pixels from the mask) were deleted from the map. The remaining tiles were kept for CNN classification (Fig. 4B). The tile size in the grid is the same as the size of a training tile (Supplementary Table 1).

Prior to processing, tiles under the mask were color-normalized using Reinhard’s method^27^ and then contextually classified by the CNN either as acinar (AC), micropapillary (MP), solid (SO), cribriform (CR) or non-tumor (NT) using the soft voting classification approach formulated as:

1 $$\hat{y}={\rm{\arg }}\,{\max }_{i}\sum _{j=1}^{N}{a}_{j}{p}_{ij}$$

where: ${p}_{ij}$ is the probability of the *j*-th tile belonging to the *i*-th tissue class $i\in \{1\ldots 5\}$, *a*_*j*_ is the weight, and *N* is the total number of tiles in the neighborhood. In our pipeline, the weights are uniform and *N*=9.

The idea of contextual classification (Equ.1) resembles a classification of a set of glimpses into the image. Then an external decision function is applied to combine the result of each glimpse. In our case, the glimpses were extracted by a fixed policy and then combined by a permutation invariant operation (soft voting), to render our final decision system (Fig. 4C). While we did not include a learnable function to inter-glimpse relationships, we nevertheless introduced the contextual classification to investigate whether it yields accurate results. Classified tiles were color coded and organized as a tumor map to overlay directly onto the original WSI for visual assessment and performance evaluation. Tiles from WSIs were imported to the pipeline through OpenSlide libraries^38^.

### Tumor growth pattern classification performance measured against pathologist ground truth

Classification performance of the four tumor growth patterns (AC, SO, MP, CB) and the non-tumor (NT) tissue was reported using 5 × 5 confusion matrices with rows representing ground truth and columns representing computed results. For a single slide, the confusion matrix was formed by superimposing the tumor map outputted by the CNN (Fig. 4C) onto a corresponding pathologist ground truth mask and then counting true positive (TP), true negative (TN), false positive (FP) and false negative (FN) pixel detections under the ground truth mask for each of the five tissue classes. For each CNN model, we generated one study-level, three set-level and 128 slide-level confusion matrices. A set-level confusion matrix was formed by concatenating all slide-level confusion matrices from a set. The three sets are CSMC, MIMW and TCGA. Further concatenation of the set-level confusion matrices into one yielded the study-level confusion matrix. F1-scores were calculated to focus the analysis on the performance in recognizing individual tumor growth patterns. To compare each model’s performance in slides irrespectively of the tumor growth pattern, size and number of annotated areas on a slide, and the slide origin, the measure of accuracy (ACC) was applied. ACC and F1-scores were calculated directly from a confusion matrix as defined previously^39,40^.

We first calculated the ACC of the models in the validation and test slides from CSMC. The models separated tissues into five classes. The validation slides (n = 19) originated from a sub-group of CSMC 14 cases that were also used for training. However, for each case the training and test slides were obtained from different paraffin blocks of the same tumor (Fig. 1). In this experiment, we investigated potential biases arising from using the same cases (but different slides) for testing and training. We queried whether classification accuracy of a CNN model is the same in the validation and test sets from CSMC. The presence of a statistically significant difference with a lower performance shown in the test set would be indicative of model overfitting. This hypothesis was tested using the Wilcoxon rank sum test. This statistical evaluation is reinforced by the fact that the test and validation and slides differed in tumor composition (Supplementary Table 2): the 19 validation slides did not contain cribriform growth pattern, and the number of annotated regions of solid growth was much higher in the test set compared to the validation set. Furthermore, for all trained models we calculated the mean ACC (5-class classification) in the combined set (n = 128) of the validation and test slides. This experiment allowed us to identify one best performing model that we compared to the FT-AlexNet which we considered the baseline model.

In the next step, using slide-level confusion matrices we calculated and then plotted distributions of the five F1-scores to rank the recognition performance for each tumor growth pattern and non-tumor tissue. ACCs were juxtaposed for comparison of slide classification accuracy in CSMC (n = 58, test and validation slides combined), MIMW (n = 43) and TCGA (n = 27) slide sets. The F1-scores and ACCs achieved by the best-performing model and the baseline FT-AlexNet were statistically evaluated (Wilcoxon signed rank test) to identify differences in classification performance between these models.

Using the study-level confusion matrix, we also calculated a F1-score for each tumor growth pattern. Subsequently, we compared the F1-scores for annotations of solid, micropapillary and cribriform growth patterns which we labeled collectively as tumor patterns associated with worse prognosis, in contrast to the F1-score for annotations of tumors with acinar growth that we labeled as a tumor pattern associated with poor prognosis. Furthermore, we merged annotations from all tumor growth patterns under one tumor category and assessed the general performance for distinguishing tumor from non-tumor tissue and provided an F1-score for each of these two categories. Lastly, we summarized the performance of the model by the overall classification accuracy for five class and two category (any tumor vs. non-tumor) tissue recognition scenarios. The F1-scores and ACC measures were calculated for the best-performing model and for the baseline FT-AlexNet using data aggregated in study-level confusion matrices.

## Results

### Evaluation of tumor growth pattern classification using pathologist annotations

**Figure 5:**
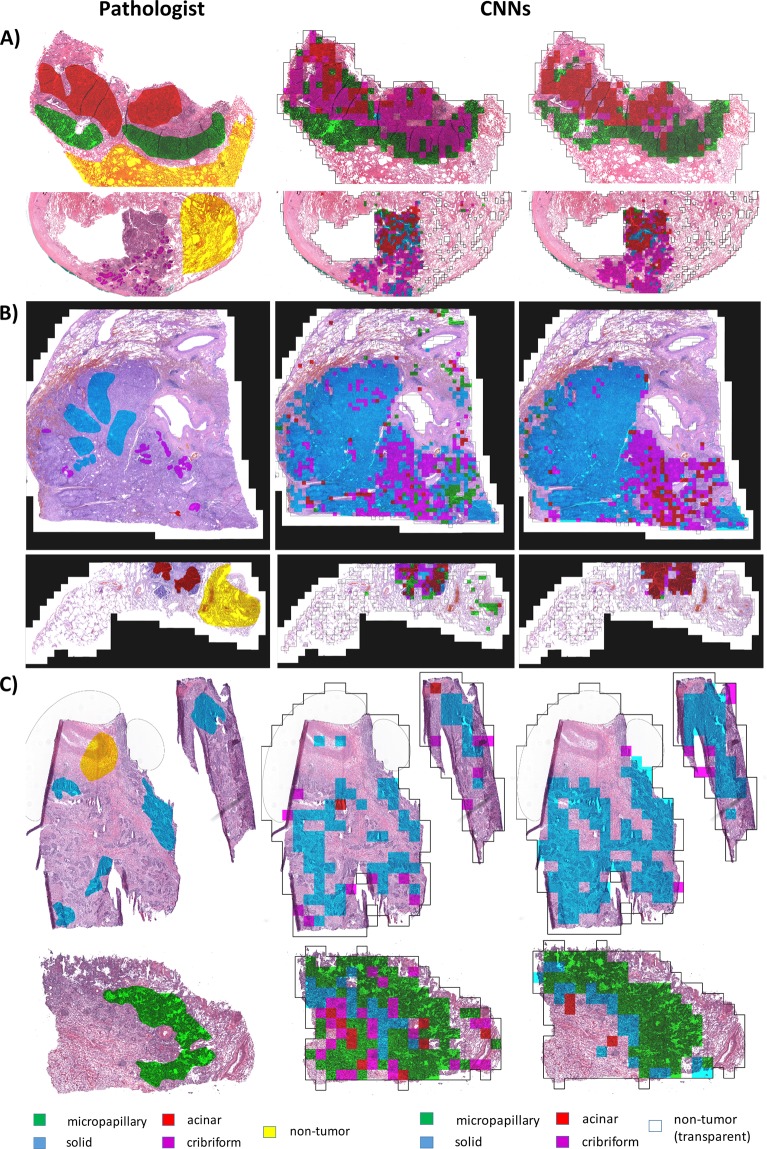
Classification of LAC tumor growth patterns in digital slides. Slides classified by CNNs are outputted as colored maps showing the growth pattern and location of solid, micropapillary, acinar, and cribriform tumor growth patterns. Middle column: maps outputted by FT-AlexNet, right column: maps outputted by DN-AlexNet. Reference annotations by pathologists are shown in the leftmost column. Example slides are from (**A**) CSMC, (**B**) MIMW and (**C**) TCGA cohorts. Pathologist’s annotations and computer-generated maps were overlaid to calculate measures of CNN performance.

Visual assessment of tumor maps outputted by our classification pipeline was performed prior to quantitative evaluation of tumor growth pattern classification results. The tumor maps were false colored and displayed side-by-side to original H&E slides with superimposed pathologist annotations (Fig. 5). Subsequently, a 5 × 5 confusion matrix representing the classification performance was calculated for each digital slide.

The Wilcoxon rank sum test was performed for one training instance of FT-AlexNet, for four of DT-AlexNet, and for three of GoogLeNet and ResNet-50 (Supplementary Table 3) to investigate the difference in classification accuracy of slides from the CSMC test and validation sets. Slides from the test set were processed with a slightly higher accuracy for each CNN instance. However, a statistically significant difference in accuracy of 3.42% (*p* = 0.045) was obtained only for one training instance of the DN-AlexNet model. All other training instances of this model and other models yielded an accuracy that was not significantly different. Prompted by the lack of robust evidence to indicate that a de-novo trained model can always output tumor maps that are significantly more accurate, we appended the CSMC validation set to the CSMC test set for further performance evaluations.

In order to select the best trained CNN, we calculated the accuracy of classifying tissues from all 128 test WSIs into five classes. One of the DN-AlexNets performed better than the best GoogLeNet and Resnet-50 CNNs which yielded ACCs that were lower by 4.06% and 2.26%, respectively (Supplementary Table 4). Thus, for the final evaluation of LAC growth pattern classification, we compared the F1-scores and ACCs obtained from the tumor maps by the best-performing DN-AlexNet trained and the FT-AlexNet model.

**Figure 6:**
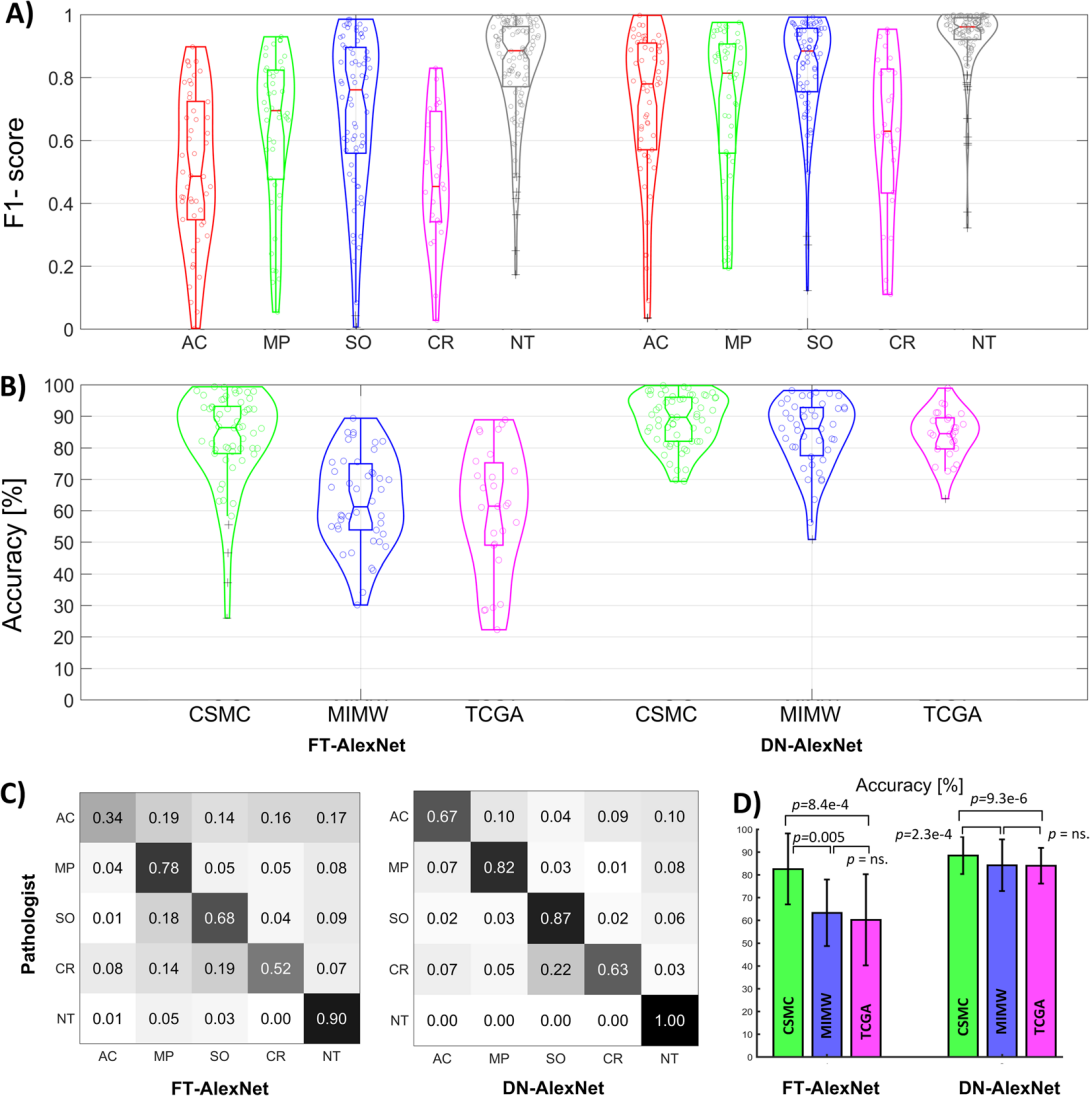
LAC tumor growth pattern classification performance in three independent sets by two models: (**A**) violin plots of F1-scores showing classification performance for acinar (AC), micropapillary (MP), solid (SO) and cribriform (CR) tumor growth patterns and non-tumor (NT) tissue in all test slides (n = 128). One data point represents one tissue class in one slide. (**B**) Violin plots of whole slide classification accuracy in slides from CSMC, MIMW and TCGA test sets. One data point represents one slide. **C**) Normalized study-level confusion matrices. Confusion matrices from CSMC, MIMW and TCGA test sets are shown in Supplementary Figure 2.

We first calculated F1-scores and accuracies using slide-level confusion matrices (n = 128). The de-novo trained model achieved significantly higher F1-scores than the fine-tuned model for all tissue classes: AC (*p* < 8.7e-7), MP (*p* < 0.002), SO (*p* < 2.e-8), CR (*p* < 0.001) and NT (*p* < 1.2e-14). The distribution of F1-scores categorized according to tumor pattern is shown in Fig. 6A. The de-novo trained model was also more accurate in the overall classification regardless of the tissue class in slides from the different cohorts: CSMC (*p* < 1.9e-4), MIMW (*p* < 1.1e-8), TCGA (*p* < 1.2e-5). Corresponding accuracy distributions are shown in Fig. 6B. The study-level confusion matrices from aggregated slide-level confusion matrices shown in Fig. 6C were used to calculate F1-scores for each tumor growth pattern (Table 2) associated with worse or poor prognosis which are: 0.924 and 0.742 for DN-AlexNet, and 0.851 and 0.465 for FT-AlexNet, respectively. F1-scores from data aggregated at the set-level (Supplementary Fig. 2) are shown in Supplementary Table 5.

**Table 2:** F1-scores for lung adenocarcinoma growth patterns and non-tumor classified by two CNN models.

Tissue class	CNN model	
	FT-AlexNet	DN-AlexNet
Acinar growth	0.465	0.742
Micropapillary growth	0.465	0.762
Solid growth	0.759	0.912
Cribriform growth	0.399	0.606
Non-tumor	0.893	0.960

When the histological growth pattern of tumors was disregarded, and all tumor patterns were bundled into one category, the F1-score representing tumor was 0.965 for the DN-AlexNet and 0.897 for the FT-AlexNet. The F1-score for non-tumor was 0.960 and 0.893 for these two models, respectively. The accuracies in classification involving the five tissue classes were 89.9% (DN-AleXNet) and 75.3% (FT-AlexNet) when all test sets from CSMC, MIMW, and TCGA were combined.

The average ACC for the CSMC, MIMW and TCGA set was 88.5%, 84.2% and 84%, respectively for the de-novo model, and 82.5%, 63.2%, and 60.2%, respectively for the fine-tuned model (Fig. 6D). The overall accuracy in the CSMC set was also significantly better than the accuracy in the MIMW and TCGA sets, irrespective of the model. The overall tumor versus non-tumor classification accuracies reached 96.1% in CSMC set and 89.9% in MIMW set.

## Discussion

Increasing evidence indicates that LAC comprises a heterogeneous group of growth patterns and that tumor growth patterns in the excised tumor specimen impact the clinical prognosis^7–9,41^. However, machine learning tools that reliably quantify the growth patterns of LAC in whole slides are currently unavailable. It is also unclear how they can learn tumor growth patterns for effective classification. To address these needs, we have developed an analytical pipeline that automatically analyzes digital slides and identifies areas of solid, microcapillary, acinar and cribriform patterns for quantification.

### Tumor growth patterns can be reliably classified in digital slides

Classification performance of the de-novo trained model was significantly better than the pretrained and fine-tuned model (Supplementary Figs 2 and 3). High F1-scores for solid (0.912), micropapillary (0.762) and acinar (0.742) growth patterns in digital slides of routinely prepared H&E stained sections of excised LAC indicated excellent ability of the de-novo trained model to distinguish one tumor growth pattern from another. The F1-score was highest for solid tumor growth in both models. Since solid tumors are composed of nests or sheets of tumor cells that lack acini and papillae, we infer that its architectural features are more straightforward to learn than the features of microcapillary, acinar, and cribriform growth which alone are more structured and heterogenous^42^ (Fig. 3). The challenge of learning complex morphologic patterns is demonstrated by only moderate recognition performance for the cribriform pattern (F1-score = 0.606) which ranked lowest amongst the four tumor growth patterns studied. Both CNN models confused this pattern mainly with the solid pattern and to a lesser extent with the other tumor growth patterns (Fig. 6C, Supplementary Fig. 2). The fine-tuned model generally underperformed except in slides with solid tumor pattern from the CSMC cohort where it achieved a slightly higher true positive detection rate than the de-novo trained model (Supplementary Fig. 2). Several studies have shown that grouping adenocarcinoma growth patterns with similar survival can strengthen the prognostic impact of the classification^2,3^. The F1-scores achieved by the de-novo trained model for tumors stratified into those associated with worse or poor outcomes were very high. The pre-trained model achieved satisfactorily high F1-scores only for the group associated with worse outcomes.

The de-novo trained model was very accurate (ACC = 96.1%) in distinguishing tumor from non-tumor areas. However, the performance of the pre-trained model was only slightly inferior (ACC = 89.92%). These accuracies are unbiased because the fractions of all tumor and all non-tumor pixels in the whole dataset are nearly the same (Fig. 2). Interestingly, both performance rates are close to those reported in studies in which more complex CNN models were trained. For instance, Arujo *et al*.^43^ distinguished regions of carcinoma from non-carcinoma in breast tumors with an accuracy of 83.3%. The same model distinguished normal tissue, benign lesion, *in situ* carcinoma and invasive carcinoma (a four-class classification) with an accuracy of 77.8%. In study by Graham *et al*.^21^ a ResNet-32 network trained on image tiles from LAC and small cell carcinoma demonstrated an 81% accuracy. To distinguish tumor from “normal tissue”, Coudray *et al*.^22^ applied a de-novo trained Inception-v3 network. However, the 96.1% accuracy achieved by this much more complex network was matched by our less-complex model. Inception-v3 trained in^19^ showed 88.1% accuracy for tumor patches and 93.5% for non-malignant patches. In another study, Khosravi *et al*.^20^ fine-tuned Inception-V1 to distinguish LAC from squamous cell carcinoma in TCGA images. Their model achieved an accuracy of 82%. Since the slides were not annotated for adenocarcinoma growth patterns, the authors recognized that this result (inferior in fact to their other classification experiments) is worsened by the heterogeneity in LACs. Although it was not the main aim of our study, our experiments with LAC showed that more complex CNNs (GoogleNet and ResNet-50) can yield a lower classification accuracy than a simpler CNN. To summarize, our best-performing CNN model matches or outperforms more complex models in recognizing tumor areas^19–22,43^. Since our best model had fewer weights to train, we suggest that its superior ability to generalize can be attributed to the quality and diversity of data collected for this experiment and used in training our models.

### Differences in classification of slides from CSMC, MIMW and TCGA sets

In contrast to CSMC slides for which classification rates were excellent, the slides from MIMW and TCGA validation sets were classified with a significantly lower accuracy (Fig. 6D). To explain this discrepancy, we inspected the quality of the slides and compared the percentages of annotated pixels in all three sets (Fig. 2). First, the tissue preservation in slides from CSMC and MIMW was superior to that in the slides from TCGA which also contained occasional tissue processing artifacts (Fig. 5C). Second, the fraction of all non-tumor pixels was higher in the CSMC slides (63%) compared to the TCGA slides (44%). Since non-tumor areas are recognized with the highest F1-score (Table 2), we assume that the superior classification accuracy of CSMC slides can be explained by better slide quality and higher proportion of non-tumor pixels in annotated areas. On average, the classification accuracy in slides from the MIMW set was also inferior to the classification accuracy of slides from CSMC, but similar to that for TCGA slides. However, slides in the MIMW set have lowest percentage of non-tumor pixels (39%), and a six times higher proportion of cribriform pixels than the other two sets. Together, these two conditions lowered the average classification accuracy of MIMW slides to the level observed for slides from the TCGA set (Fig. 2). Despite the inter and intra cohort data variabilities, the de-novo trained model performed significantly better than the pretrained model in each of the three cohorts.

### Whole slide analysis pipeline design and development

To classify tumor growth patterns, our analytical pipeline employs a striding window technique. The stride length can vary from one pixel to the window length. Pipelines that involve a single pixel stride can output continuous class probability maps and are applicable to the detection of objects ranging from single cells to large areas of tumor^44^. However, due to small stride, the analysis is slow. Pipelines that employ a stride larger than one pixel, but smaller than the window length, can analyze digital slides faster. However, they output sparse class probability maps that require computationally expensive interpolation to yield whole slide large continuous class probability maps - one for each class^22^. These approaches become even more burdensome if the number of tissue classes to be recognized is larger than two (more than just tumor vs. non-tumor classification)^21^. To optimize whole slide analysis for a project that involves recognition of 5 distinct tissue classes, we applied a striding window technique with soft voting that, in addition to the striding window, classifies eight additional overlapping tiles. No interpolation of class probability maps is performed, and the classification result is immediately saved into a ready-to-display map of tumor growth patterns for quantification (Figs 3 and 4). Considering the existing bottlenecks in the development of tools for the recognition of complex histological features in digital slides, we believe that at this stage of pipeline development the soft voting is optimal for the classification of tumor growth patterns in lung adenocarcinomas.

### Limitations and future research

The resolution of tumor map outputted by our pipeline is determined by the length of the striding window. Our pipeline currently retrieves 600 × 600 pixel tiles from the digital slide (20x mag). This size was set empirically by humans to ensure that the growth pattern in a tile of this size can be reliably assessed by expert eye. Other researchers trained their models ad-hoc on 300 × 300^19^ or 512 × 512^22^ pixel tiles at 20x magnification without providing justification in their papers. For downstream processing, our tiles need to be downsized to 256 × 256 pixels to match the receptive filed of CNNs. This solution lengthens processing time. Akin to^21^, one can retrieve 256 × 256 pixel tiles to avoid downsizing, but it remains to be tested whether this size will decrease the recognition accuracy of LAC growth patterns.

A possible improvement to our pipeline would be to implement a semantic CNN model^45^. In contrast to the originally proposed CNNs which output a class label for an image frame, its semantic brother^46,47^ can output class labels for every pixel in the frame. Application of a semantic CNN model can reduce whole slide processing time^45^. However, its training would require that fine pixel-level annotations of tumor be provided for each training tile. Since LACs are heterogenous, generating fine outlines manually for complex growth patterns such as micropapillary or acinar would be time- and cost- prohibitive. One solution to this shortcoming would be to adopt our in-house developed immunohistochemistry-based slide labeling for transferring of tumor mask from the IHC over the corresponding H&E image to obtain fine tumor delineations^48,49^.

In the current study, our models were trained to recognize four growth patterns of LAC, three of which are currently associated with worse and one that is associated with poor/intermediate prognosis^1,2,4^. A logical continuation is to add LACs exhibiting lepidic and papillary growth to the training set and to develop a model that is able to identify all six growth patterns of LACs.

## Conclusions

This is the first study to quantify tumor growth patterns in surgical specimens of lung adenocarcinoma. The tumor region maps generated by our pipeline can help pathologists quantitate total tumor area and the areas of each of four tumor growth patterns in gigapixel pathology slides. Our relatively simple CNN model was validated on slides from three separate institutions.

## Supplementary information

Supplement

Dataset 1

## Data Availability

Very few repositories offer whole slides annotated for tumor growth patterns or labeled image tiles in sufficiently large numbers to enable training of machine learning tools for digital pathology applications. Per to the review by Komura *et al*.^50^ available sets to date include data for breast^43^, prostate^45^ and colon tumors^33,51^. Our datasets would be the first pertaining to LAC and would contain one of the largest number of WSIs and image tiles for training. With the exception of the prostate cancer image repository collected previously by our team^45^, we are aware of no other repository with annotated images of tumor growth pattern. To diminish this gap, annotations for the TCGA slides used in this study are available as Supplementary Data. Software components can be found here: https://github.com/zhaoxuanma/Deeplearning-digital-pathology.
