# Supplementary material for: Convolutional neural networks can accurately distinguish four histologic growth patterns of lung adenocarcinoma in digital slides
Source: Sci Rep. 2019 Feb 6;9:1483. doi: 10.1038/s41598-018-37638-9 (PMC6365499; doi:10.1038/s41598-018-37638-9)
Supplement: Supplementary file 1 — Supplement [file 41598_2018_37638_MOESM1_ESM.docx]

**Supplementary Information**

**Convolutional neural networks can accurately distinguish four histologic growth patterns of lung adenocarcinoma in digital slides**

Arkadiusz Gertych ^1,2*^, Zaneta Swiderska-Chadaj ^3 #^, Zhaoxuan Ma^4 #^, Nathan Ing^1,4^,
Tomasz Markiewicz^3,5^, Szczepan Cierniak^5^, Hootan Salemi^1^, Samuel Guzman^2^,
Ann E. Walts^2^, and Beatrice S. Knudsen ^2,4^


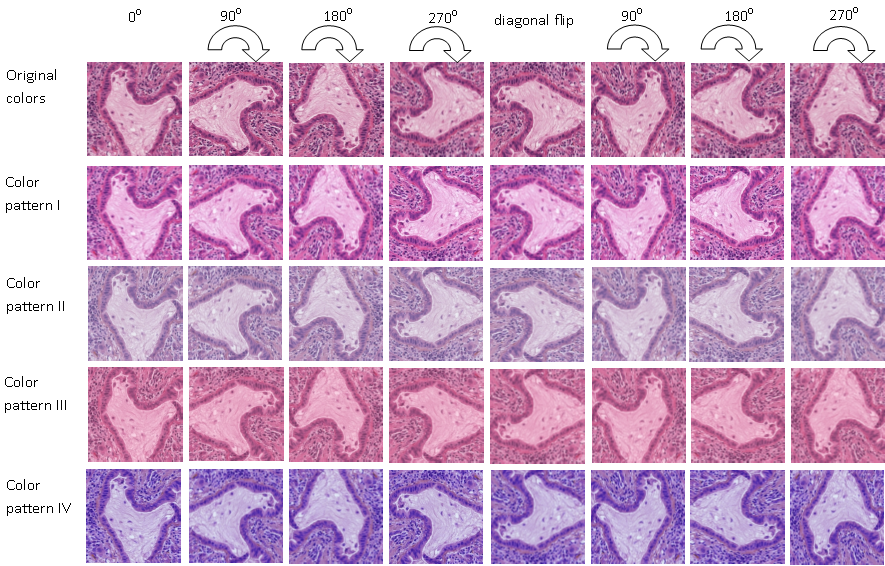


**Supplementary Figure 1**. Illustration of the image augmentation procedure. The original image is color transformed using four color patterns derived from the original data training and subsequently rotated to obtain an altered copy.


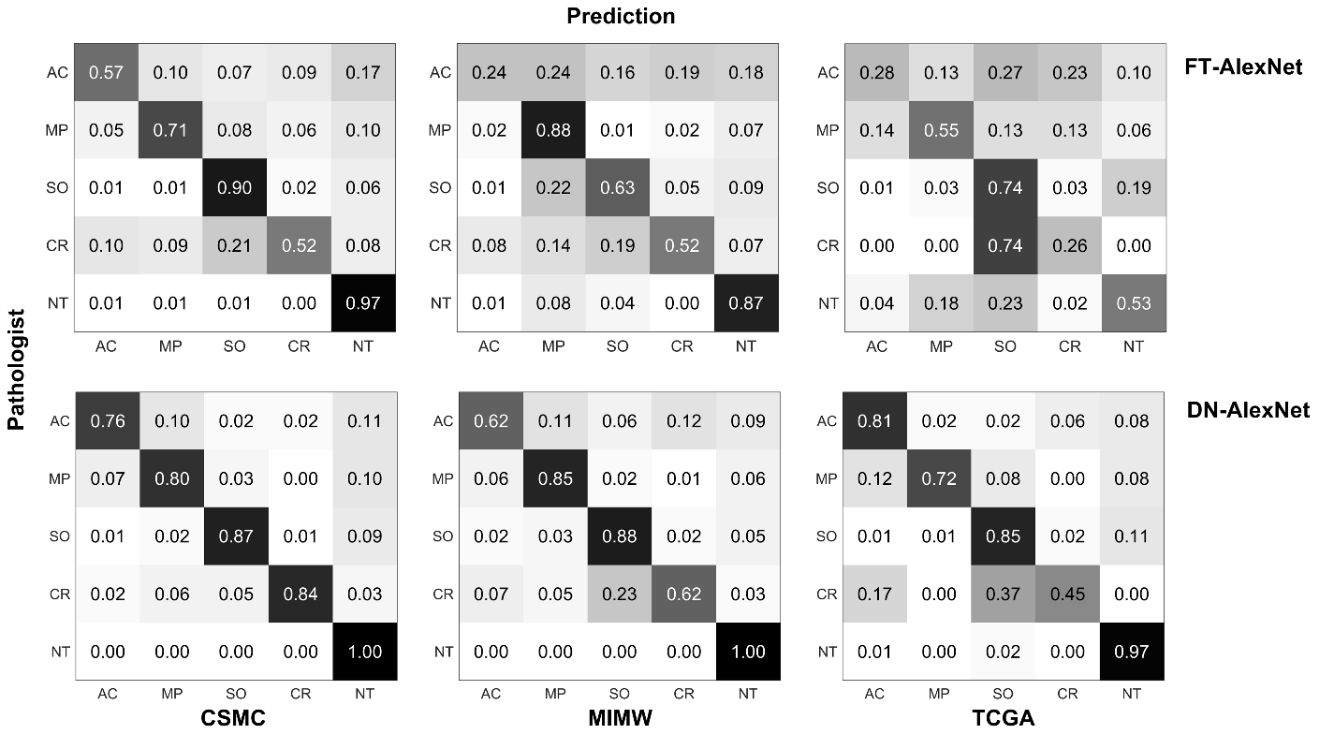


**Supplementary Figure 2**. Normalized CSMC, MIMW and TCGA cohort-level confusion matrices to calculate F1-scores for tumor growth pattern classification achieved by two CNN models.

**Supplementary Table 1.** Digital slides grouped by institution.

| Institution | CSMC | | MIMW | | TCGA | |
| --- | --- | --- | --- | --- | --- | --- |
| Set type | Training | Validation + Test | Training | Test | Test | |
| Cases total | 50 | | 33 | | 27 | |
| No. of Slides | 33 | 58 | 45 | 43 | 9 | 18 |
| WSI format | Aperio | | Mirax | | Aperio | |
| Magnification | 20x | | 20x | | 20x | 40x |
| Pixel size | 0.5μm x 0.5μm | | 0.389μm x 0.389μm | | 0.5μm x 0.5μm | 0.25μm x 0.25μm |
| Tile size (pixels) | 600 x 600 | | 756 x 756 | | 600 x 600 | 1200 x 1200 |

**Supplementary Table 2.** Number of annotated regions in validation and test sets: AC- acinar, MP- micropapillary, SO – solid, CR – cribriform, NT non-tumor.

|  | AC | MP | SO | CR | NT | Total |
| --- | --- | --- | --- | --- | --- | --- |
| CSMC validation | 55 | 30 | 63 | 0 | 56 | 204 |
| CSMC test | 57 | 45 | 108 | 184 | 105 | 499 |
| TCGA | 21 | 24 | 101 | 4 | 59 | 209 |
| MIMW | 274 | 103 | 296 | 267 | 86 | 1026 |
| Total | 407 | 202 | 568 | 455 | 306 | 1938 |

**Supplementary Table 3**. Comparison of average classification accuracy in CSMC validation and test sets. High *p*-values (Wilcoxon rank sum test) for most of the models indicate that the difference in accuracies is not statistically significant. Only one model yielded significantly different accuracies (*p*<0.05).

|  | Classification accuracy [%] | | |  |
| --- | --- | --- | --- | --- |
| CNN model | CSMC (validation) (n=19) | CSMC (test) (n=39) | Mean difference  [%] | *p*-value |
| FT-AlexNet | 84.67 | 83.00 | 1.67 | 0.420 |
| DN-AlexNet-1 | 91.66 | 89.54 | 2.12 | 0.274 |
| DN-AlexNet-2 | 91.31 | 87.49 | 3.82 | 0.329 |
| DN-AlexNet-3 | 91.82 | 89.31 | 2.51 | 0.239 |
| DN-AlexNet-4 | **93.44** | **90.02** | **3.42** | **0.045** |
| GoogLeNet-1 | 93.82 | 92.22 | 1.60 | 0.239 |
| GoogLeNet-2 | 94.29 | 91.78 | 2.51 | 0.101 |
| GoogLeNet-3 | 93.31 | 90.69 | 2.72 | 0.329 |
| Resnet-50-1 | 91.91 | 86.53 | 5.38 | 0.059 |
| Resnet-50-2 | 91.42 | 87.77 | 3.65 | 0.087 |
| Resnet-50-3 | 92.22 | 91.40 | 0.82 | 0.208 |

**Supplementary Table 4.** Classification performance in the combined set of LAC validation and test slides (n=128). The highest ACC is bolded.

| Model | Classification accuracy [%] |
| --- | --- |
| FT-AlexNet | 75.30 |
| DN-AlexNet-1 | 88.94 |
| DN-AlexNet-2 | 88.27 |
| DN-AlexNet-3 | 88.25 |
| DN-AlexNet-4 | **89.90** |
| GoogLeNet-1 | 84.52 |
| GoogLeNet-2 | 82.76 |
| GoogLeNet-3 | 85.84 |
| Resnet-50-1 | 86.89 |
| Resnet-50-2 | 86.39 |
| Resnet-50-3 | 87.64 |

**Supplementary Table 5**. F1-scores for lung adenocarcinoma growth patterns in CSMC, MIMW and TCGA sets of validation slides classified by two CNN models.

| Tissue class | CNN model | | | | | |
| --- | --- | --- | --- | --- | --- | --- |
|  | FT-AlexNet | | | DN-AlexNet | | |
|  | CSMC | MIMW | TCGA | CSMC | MIMW | TCGA |
| Acinar growth | 0.680 | 0.357 | 0.317 | 0.813 | 0.707 | 0.789 |
| Micropapillary growth | 0.751 | 0.360 | 0.500 | 0.824 | 0.709 | 0.817 |
| Solid growth | 0.888 | 0.729 | 0.662 | 0.919 | 0.912 | 0.880 |
| Cribriform growth | 0.199 | 0.432 | 0.030 | 0.625 | 0.609 | 0.181 |
| Non-tumor | 0.956 | 0.856 | 0.624 | 0.972 | 0.953 | 0.928 |
